# Determinants of quality of life among people with dementia: evidence from a South Asian population

**DOI:** 10.1186/s12877-022-03443-3

**Published:** 2022-09-12

**Authors:** Surangi Jayakody, Carukshi Arambepola

**Affiliations:** 1 Department of Community Medicine, Faculty of Medical Sciences, University of Sri Jayewardenepura, Nugegoda, Sri Lanka; 2 Department of Community Medicine, Faculty of Medicine, University of Colombo, Colombo, Sri Lanka

**Keywords:** QOL, Dementia, Determinants, South-Asian

## Abstract

**Background:**

Dementia has become a public health priority along with population aging worldwide. Owing to its chronic progressive nature in the absence of a cure, maintaining the best possible quality of life (QOL) has become the desired outcome for people with dementia (PWD).

**Aim:**

The current study aimed to determine the factors associated with ‘good QOL’ in people with dementia in the Sri Lankan setting.

**Methods:**

An unmatched case–control study was conducted to identify the factors associated with ‘good QoL. Cases in the study included dementia patients having ‘good’ QOL, whereas controls were those having ‘poor’ or ‘average’ QOL. Both cases and controls were selected from the same patient base at a premier tertiary care state hospital in Colombo District, Sri Lanka. They were identified using the validated DEMQOL (Dementia Specific Quality of Life) tool, adhering to strict eligibility criteria. An interviewer-administered questionnaire was used to assess the associated factors. Bivariate analysis followed by logistic regression modelling determined the associated factors for ‘good QOL’ adjusted for confounders using odds ratio (OR) and 95% confidence interval (CI).

**Results:**

The study sample consisted of 64 cases and 208 controls. After adjusting for confounders, education up to GCE O/Level and above (OR = 4.02; 95% CI = 2.97, 12.0), ever employed (OR = 3.21; 95% CI = 1.59, 11.06), good social functioning (OR = 4.14; 95% CI = 3.39, 16.46), mild functional impairment (OR = 1.77; 95% CI = 1.13, 9.67), little or no caregiver burden (OR = 2.96; 95% CI = 1.86, 10.94), absence of apathy (OR = 2.22; 95% CI = 1.27, 12.48) and absence of irritability (OR = 2.17; 95% CI = 1.72, 10.34) were found to be significantly associated with ‘good QOL’. 60% of the variance of ‘good’ QOL among PWD was explained by the factors in the final model.

**Conclusions and recommendations:**

The identified determinants of ‘good QOL’ clearly show how the QOL improvement interventions need to be planned. Accordingly, such programmes should be primarily focused on strategies to improve PWDs' ADL (Activities of Daily living), reduce and manage neuropsychiatric symptoms effectively and to promote activities enhancing social functioning, and plan programmes to address caregiver burden.

**Supplementary Information:**

The online version contains supplementary material available at 10.1186/s12877-022-03443-3.

## Introduction

Disease-specific quality of life (QOL) is an important outcome measure, particularly in degenerative disorders that are progressive, as in dementia where opportunities for long-term health outcomes such as symptom reduction and increased survival are limited.

There is strong evidence to suggest that the QOL of patients declines with time, as dementia progresses. Concurrently, the QOL related to dementia is generally poor across regions and different populations [1–3]. Despite this general tendency, patients at the same stage of disease appear to differ in their QOL, suggesting that there could be other modifiable factors playing a crucial role in determining their QOL [4]. Such disease-specific assessments in QOL are important for planning of service provision and as a quality indicator of the care [5]. Which in turn could minimize the impact on morbidity and socio-economic status among the PWD [1, 2, 6].

It is shown that dementia could affect the QOL of patients in different ways, depending on the impact of the disease, care received, as well as their personality before developing the disease. Lower QOL has been strongly associated with depression and anxiety in dementia [3], in contrast to higher QOL demonstrated in patients having fewer depressive symptoms, irritability, and apathy [7]. Dementia patients also report unique problems with their mobility and social support [8], which could in turn affect their QOL, whereas better QOL correlates more strongly with social interaction [7, 9], better general health [10, 11], and reduced caregiver burden [12]. Recognizing and addressing these determinants are expected to shape the current recommendations made on the long-term management of dementia patients. This has major implications in low- and middle-income countries (LMIC), where providing care for dementia patients on one hand is a novel experience along with recent escalations in population aging in such countries, and on the other, is an expensive task for individual families amidst poorly established social support schemes. However, the currently identified determinants of dementia-specific QOL have been predominantly explored in high-income countries [1, 2] and therefore unlikely to be directly applicable to LMIC especially in South Asia, owing to the regional differences related to socio-cultural backgrounds and the availability of care plans.

Sri Lanka is a South Asian country, which has become one of the fastest aging countries in the world. Its share of the population over 60 years of age, which was 12% in 2014, has exceeded the average of all countries in the South-East Asian region. According to population projections, this proportion will reach 28.5% by 2050 [13]. With ageing as the major non-modifiable risk factor for dementia, an epidemic of dementia is a certainty in Sri Lanka [14]. According to an epidemiological study done on dementia in semi urban and sub-urban populations in Sri Lanka, the prevalence of dementia was found to be 4%. In comparison with studies conducted in the region, this prevalence is much higher [15]. Despite having a well-established health care system in Sri Lanka almost in par with developed countries, it is not geared to handle medical and social issues of rapidly expanding elderly people and notably that of dementia patients. This situation is worsened by the scarcity of knowledge based on research, on the current status of patients with dementia.

To this end, the first step would be to identify the determinants of ‘good QOL’ in patients with dementia. This will provide strong evidence for policymakers to advocate effective measures that will assist in developing tailor-made interventions to improve the QOL of patients living with dementia. Hence, a case–control study was designed to determine the non-pharmacological factors for ‘good’ QOL among people with dementia in Sri Lanka.

## Methods

A hospital-based, unmatched case–control study was conducted among PWD followed-up in the premier tertiary care state hospitals in Colombo District, Sri Lanka from September 2017 to March 2018. These hospitals located in the most developed district in the country, are well-equipped with tertiary care health facilities, including specialists in neurology and psychiatry whose clinical expertise is sought in the diagnosis of dementia as well as in their follow-up. Thus, to achieve the highest patient yield, tertiary care hospitals in Colombo District were selected.

Patients' dementia-specific QOL was determined using the DEMQOL tool, which had been validated and available in local languages to assess the QOL from patient perspectives [16]. DEMQOL overall scores of the PWD were converted to Z scores and then transformed into a norm distribution, named ‘scaled scores’ with a mean of 10 and SD of 3. The cut-off value was set at 12 (75^th^ percentile) and patients with scaled scores higher than this value were considered as having ‘good’ QOL; scores less than 12 were categorized as having ‘poor’ QOL [16].

Cases in the study included dementia patients having ‘good’ QOL, whereas controls were those having ‘poor’ or ‘average’ QOL. Both cases and controls were selected from the same patient base. All of them had been followed up at least for six months in outpatient psychiatric clinics, and their diagnosis was made by a consultant psychiatrist or neurologist according to the DSM-IV criteria. The study confirmed the diagnosis based on documental evidence (e.g. diagnosis card, patient clinic record).

Patients with severe dementia (diagnosed clinically by a consultant psychiatrist and confirmed by Mini-Mental State Examination (MMSE) score less than 10 [17], institutionalized patients (e.g. elderly homes), patients with speech or hearing impairment, or any other diagnosed psychiatric condition which could compromise their ability to participate in an interview were excluded. Also, patients not accompanied by their primary caregiver (defined as the person who takes the primary responsibility of the patient and being there with him/her at least during the preceding six months) to the hospital were excluded. The sample size was calculated according to the formula on multiple unmatched controls per case [18]. Since cases (‘good’ QOL) among dementia patients were assumed to be a rare occurrence in low-income settings, multiple controls per case (3.5:1) were considered.

A pre-tested interviewer-administered questionnaire was used to assess the potential factors that influence the QOL in dementia, namely demographic and socio-economic characteristics of PWD and caregivers (age, sex, marital status, religion, ethnicity, family income, level of education, social class) and treatment-related factors such as time since diagnosis/ clinic follow up, number. of clinic visits for last 6 months and the average distance from home to clinic etc. In addition, tools already validated for Sri Lanka were used for the assessment of severity of dementia using MMSE (Mini Mental State Examination [19], Medical co-morbidities of the patient based on GMHR (General Medical Health rating) [20], Social functioning of the PWD based on SF-DEM (Social-Functioning Dementia) scale [21], Activities of daily living using ADLQ (Activities of Daily Living questionnaire) [22], and neuropsychiatric symptoms in the patient using NPI (Neuropsychiatric Inventory) [23] and Caregiver burden using Zarit Burden Interview (ZBI) [24]. The judgemental validity of the questionnaire was assessed by a panel of experts and the target population. Four trained pre-intern medical graduates did the data collection. The reliability of questionnaire completion was assessed using test–retest reliability measures. A correlation coefficient (*r*) value of 0.7 or more was considered a ‘good’ level of agreement between the scores.

### Data analysis

The factors associated with’good’ QOL were estimated in relation to the study outcomes (‘poor’ and ‘good QOL) and potential risk factors among PWD, using odds ratio (OR) and 95% confidence interval (95% CI). The significance was assessed using the Chi-squared test. Logistic regression analysis was performed to adjust for confounders, in which the factors significant in the bivariate analysis were included as independent variables in the model. All the variables except age were included as categorical type data and their reference category was identified by the lower percentage of exposure among cases. Model building was done using the backward logistic regression (LR) procedure with a probability of the variables fixed for entry at *p* = 0.05 and removal at *p* = 0.1 levels of significance. The goodness of fit of the overall model was assessed using Hosmer–Lemeshow goodness of fit test (H–L GOF), Nagelkerke R^2^ statistic and by Omnibus test at 0.05 significance level. Logistic regression coefficients (β coefficients) obtained for each variable were assessed using Wald statistic for significance and interpreted as adjusted OR and 95% CI.

## Results

The study sample consisted of 64 cases and 208 controls. The response rate was 100%. All 272 eligible pairs agreed to participate in the study after giving informed written consent without any obligation. A comparison of the basic characteristics of cases and controls is given in Table 1.

**Table 1:** Frequency distribution of the demographic characteristics of cases and controls

Demographic characteristic	QOL status			
	**Cases (*n* = 64)**		**Controls (*n* = 208)**	
	**No**	**%**	**No**	**%**
**Age (in years)**				
65 or less	11	17.3	32	15.4
66 – 75	35	53.8	112	53.8
Over 75	18	28.8	64	30.8
**Sex**				
Male	33	51.9	84	40.4
Female	31	48.1	124	59.6
**Current marital status**				
Married	37	57.7	110	52.9
Unmarried	10	15.4	30	14.4
Widowed	17	26.9	68	32.7
**Ethnicity**				
Sinhalese	60	94.2	186	89.4
Tamil	1	1.9	8	3.8
Moor	3	3.8	14	6.7
**Religion**				
Buddhism	50	78.8	170	81.7
Christianity/Catholic	10	15.4	19	9.1
Hindu	0	0	5	2.4
Islam	4	5.8	14	6.7
**Main caregiver’s relationship to patient**				
Spouse	26	40.4	65	31.2
Son/daughter	26	40.4	93	44.7
Sibling	7	11.5	31	14.9
Relative	1	1.9	7	3.4
Other/ In-laws	4	5.8	12	5.8

As shown in Table 2, 17 variables were assessed on their association with ‘good’ QOL in the bivariate analysis, of which only 13 were shown to be significant (Refer to supplementary tables for further detail).

**Table 2:** Summary of the list of associated factors included in the multiple logistic regression model

Factors	Unadjusted OR	95% CI	Significance
Age	0.86	(0.38–1.95)	0.734
Male sex	1.59	(0.86–2.93)	0.132
Spouse being the CG	1.49	(0.79–2.78)	0.210
Son/daughter being the CG	1.62	(0.97- 3.10)	0.070
Studied up to GCE O/L and/or above	4.84	(2.61–9.00)	< 0.001
Monthly income of > 20,000 rupees	3.57	(2.16–5.90)	< 0.001
Ever employed	3.36	(2.67–7.47)	< 0.001
Social class middle and above	5.63	(2.20–10.78)	< 0.001
Good general medical health	2.42	(1.17- 4.99)	0.014
Good social functioning/participation	4.55	(2.68- 7.72)	< 0.001
Mild functional impairment	2.07	(1.02- 4.19)	0.039
Absence of depressive symptoms	2.45	(1.32- 4.55)	0.004
Absence of apathy	2.86	(1.52- 5.85)	< 0.001
Absence of irritability	2.58	(1.51 -4.41)	< 0.001
Absence of disinhibition	2.23	(1.24- 8.77)	0.007
Absence of night-time behaviour	1.98	(1.44- 3.43)	0.020
Little or no caregiver burden	3.13	(1.18–8.298)	0.016

Epidemiological approach was used for the purpose of model building; the independent variables to be entered in the LR model were decided not only based on statistical significance (*p* value < 0.05 in bivariate analysis), but also considering the importance to the research outcome based on literature and expert opinion. Therefore, 17 independent variables which were selected according to the above criteria were included in the LR model.

Only 7 variables were retained in the final model as independent variables associated with good’ QOL in patients. Table 3 summarizes the strength of association of these variables.

**Table 3:** Independent variables of ‘good QOL’ among patients with dementia and their significance

Associated factor	ß	S.E	Wald	df	Sig	Exp.ß	95% CI for Exp.ß	
							**Lower**	**Upper**
Studied up to GCE O/L and/or above	2.11	.523	6.39	1	.000	4.02	2.97	18.09
Ever employed	1.65	.605	7.44	1	.006	3.21	1.59	15.06
Good social functioning/participation	2.18	.493	9.67	1	.000	4.14	3.39	19.43
Mild functional impairment	1.13	.561	4.06	1	.014	1.77	1.13	9.67
Absence of apathy	2.19	.657	8.99	1	.001	2.22	1.27	16.48
Absence of irritability	2.32	.539	5.95	1	.000	2.17	1.72	15.34
Little or no caregiver burden	1.24	.687	3.14	1	.016	2.96	1.86	10.94

Reference categories are not shown in the table

Out of the socio-economic characteristics, PWD who had studied up to GCE O/Level and /or above were four times more likely to have ‘good QOL’ (adjusted OR = 4.02; 95% CI = 2.97, 18.09) compared to those who had studied up to grade 10 or below (*p* < 0.001). Further, PWD who were ever employed were 3.21 times more likely to have ‘good QOL’ (adjusted OR = 3.21; 95% CI = 1.59, 15.06) compared to those who were never employed (*p* = 0.006). However, monthly income of > 20,000 rupees and social class middle and above did not show significant associations with ‘good QOL’ after adjusting for confounding. PWD who were having good social functioning/participation were 4.14 times more likely to have ‘good QOL’ (adjusted OR = 4.14; 95% CI = 3.39, 19.43) compared to those who were having poor social functioning (*p* < 0.001).

With regards to functional activities of daily living, those having only mild impairment were 1.77 times more likely to have ‘good QOL’ (adjusted OR = 1.77; 95% CI = 1.13, 9.67) compared to those having a severe functional impairment (*p* = 0.01).

Of the neuropsychiatry symptoms, PWD who did not show symptoms suggestive of apathy (adjusted OR = 2.22; 95% CI = 1.27, 16.48) and irritability (adjusted OR = 2.17; 95% CI = 1.72, 15.34) were about 2 times more likely to have ‘good QOL’ compared to the corresponding categories (*p* =  < 0.001).

When the caregiver burden was considered, dementia patients whose caregivers showed little or no burden were 2.96 times more likely to have ‘good QOL’ (adjusted OR = 2.96; 95% CI = 1.86, 10.94) compared to those having caregivers with a high burden (*p* = 0.02).

According to the Omnibus tests of model coefficients, the final model showing a model chi-square of 125.279 with 11 df, was statistically significant at *p* < 0.001 level. The H–L GOF test chi-square value for the final model was 2.160 (df = 8) with a non-significant *p* value of 0.976. Nagelkerke R^2^ statistic of the final model was 0.605, indicating that 60% of the variance of ‘good QOL’ among patients with dementia was explained by the factors in the final model.

## Discussion

In the current study, demographic factors, namely age of the patient and caregiver, sex, marital status, religion, and caregiver’s relationship with the patient did not show significant associations with ‘good’ QOL. This is consistent with most of the available literature [3, 7, 9]. In contrast, all the socio-economic factors studied showed significant associations with ‘good’ QOL in the univariate analysis. Of these, higher level of education and ever been employed remained significant after adjusting for confounders. This may be a reflection of their socio-economic stability or social functioning related to employment. A study done in Thailand showed a similar association between poor QOL and low level of education (*p* = 0.004) [25]. In contrast, most of the studies done in developed countries had not shown such significant associations with socio-economic factors [1, 7, 26]. In developed countries, employment status may not be playing a major role in the QOL in dementia, as there are well-established social care services tailor-made for dementia patients that penetrate through all layers of the society and help in minimising the social gaps.

There was no significant difference in QOL according to the severity of dementia in the current study. This is consistent with most of the available literature [1, 12, 26]. It should however be noted that these studies, including the current study, have not incorporated those with severe dementia. Therefore, this association may not hold true for patients with severe dementia.

A meta-analysis conducted by Martyr et al. [7] on 31 empirical studies found co-morbid conditions to be significantly associated with poor QOL (*p* < 0.001). In comparison, better general health was significantly associated with ‘good’ QOL in the current study (OR = 2.42; 95% CI = 1.17, 4.99; *p* = 0.01) but its significance was lost when adjusted for confounders. Nevertheless, this can be considered an important finding as the patient’s general medical health status is modifiable in a positive manner with proper clinical management. In Sri Lanka, especially at tertiary care level, almost all psychiatry clinics are conducted under the purview of a consultant psychiatrist. In these clinics, dementia patients are also assessed for co-morbid medical conditions and referred if required. Furthermore, the absence of apathy and irritability were significantly associated with ‘good’ QOL among dementia patients. The results were consistent with several studies done in developed countries [3, 10, 12]. These findings warrant the need for comprehensive assessments of patients with dementia in relation to psychological status.

Patients with good social functioning were four times more likely to have ‘good’ QOL (adjusted OR = 4.14; 95% CI = 3.39, 19.43). There is strong research evidence from around the world to support this association. A meta-analysis (Martyr et al., [7]) found greater social engagement to be significantly correlated with QOL (*r* = 0.31; 95% CI = 0.12, 0.48; *p* = 0.02) and a scoping review (Holopainen et al., [9]) found social functioning as a factor associated with better QOL. A meta-synthesis reviewing qualitative studies on dementia QOL and associated factors also supports this finding as ‘social connectedness’ was a key theme emerged from patients and their caregivers [27].

With regards to functional activities of daily living, those having only mild impairment of ADL were 1.77 times more likely to have ‘good’ QOL (adjusted OR = 1.77; 95% CI = 1.13, 9.67) compared to others (*p* = 0.01). This result is compatible with a systematic review (Martyr et al., [7]) showing better functional ability as a significant factor associated with QOL (*r* = 0.27; 95% CI = 0.17, 0.32; *p* < 0.001).

When the caregiver burden was considered, dementia persons with caregivers who showed little or no burden were 2.96 times more likely to have ‘good’ QOL (adjusted OR = 2.96; 95% CI = 1.86, 10.94) compared to others with high caregiver burden (*p* = 0.02). This is in line with other available research evidence. Woods et al. [12] showed a significant negative correlation between caregiver distress and QOL (*r* = -0.33; *p* < 0.001). A cross-sectional study done among dementia patients living in residential care facilities in Australia showed a significant association of caregiver distress with poor QOL (*p* < 0.001) [28].

In most of the developed countries, frail elderly persons including patients with dementia frequently receive their multi-dimensional assessment from a social worker or a nurse who is defined as a ‘care manager’ or a ‘care coordinator’. These assessments are typically performed at the patient’s own home using a structured schedule, which includes an assessment of the basic and instrumental activities of daily living and also an assessment of social functioning. The physician customizes the individual patient’s management plan according to the findings [29]. Despite strong evidence on social functioning and ADL being helpful to enhance a person’s QOL to a great extent, there is currently no established home-based assessment programme for dementia patients in Sri Lanka. However, these initiatives need to be more organized and incorporated into the routine patient management system.

## Limitations

Although a longitudinal study design would have been more appropriate to assess the temporal relationship of the factors associated with ‘good QOL’, a case–control design had to be used due to the smaller number of available patients and resource constraints. Cases and controls were identified based on validated DEMQOL tool; however, there could still be misclassification bias. Several tools were used to assess the associated factors such as MMSE, NPI, ADLQ, SF-DEM and ZBI. All these tools underwent translation into local languages and cultural adaptation. However, assessing their construct validity was out of scope of the current study. Study was based on tertiary care state hospital psychiatry clinic follow up patients in Colombo district. The participants were selected adhering to strict inclusion /exclusion criteria to maximize the internal validity. Hence, the results may not be generalized to all the dementia patients in the country owing to the selective sample obtained.

## Conclusions and recommendations

The identified associated factors with ‘good QOL’ clearly show how the QOL improvement interventions need to be planned. Accordingly, such programmes should be primarily focused on strategies to improve patients' ADL, to reduce and manage neuropsychiatric symptoms effectively and to promote activities enhancing social functioning, and to plan programmes to address caregiver burden.

Proper functional assessment by health care professionals is recommended in every patient with dementia at baseline and at regular intervals. Activities to improve ADL should be implemented and family members should be educated regarding the importance of ADL in limiting the progression of disease and improving QOL.

## Supplementary Information

**Additional file 1.**

## Data Availability

The raw datasets used during the current study are not publicly available due to ethical reasons but are available from the corresponding author upon reasonable request. However, all analyzed data are included in this manuscript and its supplementary file.

## Abbreviations

ADL: Activities of daily living
ADLQ: Activities of Daily living questionnaire
CI: Confidence Interval
DEMQOL: Dementia Quality of life
GCE O/L: General Certificate of Education Ordinary Level
H–L GOF: Hosmer–Lemeshow goodness of fit test
LMIC: Low- and middle-income countries
MMSE: Mini-Mental State Examination
NPI: Neuropsychiatric Inventory
OR: Odds ratio
PWD: People with dementia
QOL: Quality of life
SF-DEM: Social Functioning in Dementia
ZBI: Zarit Burden Interview
