# Supplementary material for: Determinants of quality of life among people with dementia: evidence from a South Asian population
Source: BMC Geriatr. 2022 Sep 12;22:745. doi: 10.1186/s12877-022-03443-3 (PMC9469587; doi:10.1186/s12877-022-03443-3)
Supplement: Supplementary file 1 — Additional file 1. [file 12877_2022_3443_MOESM1_ESM.docx]

**Supplementary data**

***Table 1: Frequency Distribution of the Demographic Characteristics of Cases and Controls***

| **Demographic characteristic** | **QOL status** | | | | |
| --- | --- | --- | --- | --- | --- |
|  | **Cases (n=64)** | | **Controls (n=208)** | | |
|  | **No.** | **%** | | **No.** | **%** |
| **Age (in years)** |  |  | |  |  |
| 65 or less | 11 | 17.3 | | 32 | 15.4 |
| 66 – 75 | 35 | 53.8 | | 112 | 53.8 |
| Over 75 | 18 | 28.8 | | 64 | 30.8 |
| **Sex** |  |  | |  |  |
| Male | 33 | 51.9 | | 84 | 40.4 |
| Female | 31 | 48.1 | | 124 | 59.6 |
| **Current marital status** |  |  | |  |  |
| Married | 37 | 57.7 | | 110 | 52.9 |
| Unmarried | 10 | 15.4 | | 30 | 14.4 |
| Widowed | 17 | 26.9 | | 68 | 32.7 |
| **Ethnicity** |  |  | |  |  |
| Sinhalese | 60 | 94.2 | | 186 | 89.4 |
| Tamil | 1 | 1.9 | | 8 | 3.8 |
| Moor | 3 | 3.8 | | 14 | 6.7 |
| **Religion** |  |  | |  |  |
| Buddhism | 50 | 78.8 | | 170 | 81.7 |
| Christianity/Catholic | 10 | 15.4 | | 19 | 9.1 |
| Hindu | 0 | 0 | | 5 | 2.4 |
| Islam | 4 | 5.8 | | 14 | 6.7 |
| **Main caregiver’s relationship to patient** |  |  | |  |  |
| Spouse | 26 | 40.4 | | 65 | 31.2 |
| Son/daughter | 26 | 40.4 | | 93 | 44.7 |
| Sibling | 7 | 11.5 | | 31 | 14.9 |
| Relative | 1 | 1.9 | | 7 | 3.4 |
| Other/ In-laws | 4 | 5.8 | | 12 | 5.8 |

***Table 1.2: Frequency Distribution of the Socioeconomic Characteristics of Cases and Controls***

| **Socio-economic characteristic** | **QOL status** | | | |
| --- | --- | --- | --- | --- |
|  | **Cases (n=64)** | | **Controls (n=208)** | |
|  | **No.** | **%** | **No.** | **%** |
| **Level of education** |  |  |  |  |
| Grade 1-5 | 1 | 1.9 | 52 | 25 |
| Grade 6 -10 | 12 | 19.2 | 84 | 40.4 |
| Passed G.C.E O/Level | 19 | 28.8 | 51 | 24.5 |
| Passed G.C.E A/Level | 26 | 40.4 | 15 | 7.2 |
| Degree /Diploma | 6 | 9.6 | 6 | 2.9 |
| **Monthly income (in Rupees)** |  |  |  |  |
| No permanent income | 1 | 1.9 | 40 | 19.2 |
| 10,000 or less | 0 | 0 | 42 | 20.2 |
| 10,001 – 20,000 | 23 | 34.6 | 74 | 35.6 |
| 20,001 – 30,000 | 39 | 61.5 | 45 | 21.6 |
| More than 30,000 | 1 | 1.9 | 7 | 3.4 |
| **Occupational category (past/present)** |  |  |  |  |
| Administrative and Managerial | 0 | 0 | 0 | 0 |
| Professional/ Technical and related | 5 | 7.7 | 0 | 0 |
| Clerical and related | 22 | 34.6 | 7 | 3.4 |
| Sales workers | 2 | 3.8 | 23 | 11.1 |
| Supervisors including Foreman | 14 | 21.2 | 11 | 5.3 |
| Skilled/ Semi-skilled | 15 | 23.1 | 48 | 23.1 |
| Unskilled | 0 | 0 | 19 | 9.1 |
| No occupation | 6 | 9.6 | 100 | 48.1 |
| **Social class** |  |  |  |  |
| Low | 1 | 1.9 | 60 | 28.8 |
| Middle | 60 | 94.2 | 148 | 71.2 |
| High | 3 | 3.8 | 0 | 0 |

***Table 4: Frequency Distribution of the Severity of Dementia in Cases and Controls***

| **Dementia severity** | **Total** | **QOL status** | | | |
| --- | --- | --- | --- | --- | --- |
|  |  | **Cases (n= 64)** | | **Controls (n=208)** | |
|  |  | **No.** | **%** | **No.** | **%** |
| Mild (>18MMSE score) | 171 | 42 | 65.4 | 137 | 65.9 |
| Moderate (Less than 18 MMSE score) | 89 | 22 | 34.6 | 71 | 34.1 |

**General medical health of the patients with dementia**

The medical co-morbidity of dementia patients was assessed by the interviewers using the General Medical Health Rating Scale (GMHR) ([Lyketsos et al., 1999](#_ENREF_7)). GMHR is a ‘global clinical rating’ which quantifies the severity of general co-morbidities in a patient with dementia as a single number. Table 5 describes the medical co-morbidities among cases and controls.

***Table 5: Frequency Distribution of the General Medical Health of Cases and Controls***

| **General medical health of patients** | **QOL status** | | | |
| --- | --- | --- | --- | --- |
|  | **Cases (n=64)** | | **Controls (n=208)** | |
|  | **No.** | **%** | **No.** | **%** |
| Poor | 0 | 0 | 2 | 1.0 |
| Fair | 13 | 21.2 | 80 | 38.5 |
| Good | 32 | 50.0 | 113 | 54.3 |
| excellent | 19 | 28.8 | 13 | 6.2 |

***Table 6: Frequency Distribution of the Social Functioning of Cases and Controls***

| **Social Functioning components** | **QOL status** | | | | | | | | | | |
| --- | --- | --- | --- | --- | --- | --- | --- | --- | --- | --- | --- |
|  | **Cases (n=64)** | | | | | **Controls (n=208)** | | | | | |
|  | **Mean (SD)** | | **95% CI** | | **Range** | | **Mean (SD)** | | **95% CI** | | **Range** |
| ‘current social activities’ | 9.82  (3.68) | 8.8-10.85 | | 3 , 15  (12) | | 7.09  (3.97) | | 6.55-7.63 | | 2, 15  (13) | |
| ‘Personal relationships’ | 11.78 (3.17) | 10.9-12.67 | | 5, 15  (10) | | 10.22  (2.63) | | 9.86-10.58 | | 2, 12  (10) | |
| **Total score** | **21.61**  **(2.96)** | **20.79-22.44** | | **13, 25**  **(12)** | | **17.31**  **(3.71)** | | **16.80-17.82** | | **11, 26**  **(15)** | |

***Table 6.1: Frequency Distribution of the Overall Social Functioning (Based on Caregiver Responses) of Cases and Controls***

| **Overall social functioning based on caregiver responses** | **QOL status** | | | |
| --- | --- | --- | --- | --- |
|  | **Cases (n=64)** | | **Controls (n=208)** | |
|  | **No.** | **%** | **No.** | **%** |
| **1. Thinking about their social life as a whole how it is now?** | | | | |
| Good | 34 | 53.8 | 56 | 26.9 |
| Fair | 26 | 40.4 | 84 | 40.4 |
| Poor | 4 | 5.8 | 68 | 32.7 |
| **2. How is it now compared to one year ago?** | | | | |
| No change | 22 | 34.6 | 107 | 51.4 |
| A bit worse | 42 | 65.4 | 100 | 48.1 |
| A lot worse | 0 | 0 | 1 | 0.5 |
| **3. Would you like their social life to change?** | | | | |
| Rather do more | 58 | 90.6 | 190 | 91.3 |
| No change needed | 6 | 9.6 | 18 | 8.7 |

Most of the caregivers of cases perceived that their patient’s social life was ‘good’(53.8%) while only 5.8% stated it as ‘poor’, whereas the majority of caregivers of the controls perceived their patient’s social life as ‘fair’(40.4%) and 32.7% as ‘poor’.

**Activities of daily living of the dementia patients**

The activities of daily living of the dementia patients were assessed using the Activities of daily Living Questionnaire (ADLQ ([Johnson et al., 2004](#_ENREF_5)). ADLQ is a caregiver-based assessment of functional abilities in patients with dementia. The ADLQ measures functioning in six areas: self-care, household care, employment and recreation, shopping and money, travel and communication. Each section has three to six items, which are rated on a four-point Likert scale. The total score which represents the severity of impairment was calculated using a formula specified by the original authors of the tool. The scores ranged from 0 to 100. The higher the score, the more severe the impairment was.

The functional impairment of patients with dementia was categorized as follows;

- Total score 0-33 = ‘None to mild’ impairment of ADL
- Total score 34-66 = ‘Moderate’ impairment of ADL
- Total score > 66 = ‘Severe’ impairment of ADL

Table 7 summarizes the average ADL scores among cases and controls.

***Table 7: Average Sub-component and Total Scores of Activities of Daily Living (ADL) among Cases and Controls***

| **ADL**  **subscales** | **QOL status** | | | |
| --- | --- | --- | --- | --- |
|  | **Cases** | | **Controls** | |
|  | **Mean (SD)** | **95% CI** | **Mean (SD)** | **95% CI** |
| Self-care | 3.62 (2.49) | 2.92- 4.31 | 7.24(5.02) | 6.55 -7.93 |
| Household | 9.17(3.76) | 8.12 - 10.22 | 11.44 (4.44) | 10.83-12.05 |
| Employment | 6.08 (2.48) | 5.39- 6.77 | 6.88 (3.55) | 6.40-7.37 |
| Shopping | 6.85 (2.85) | 6.05-7.64 | 7.26 (2.25) | 6.96 -7.57 |
| Travel | 5.08 (2.67) | 4.33-5.82 | 8.47 (3.08) | 8.06 -8.88 |
| Communication | 6.58 (2.68) | 5.65-7.50 | 7.00 (4.37) | 6.4 -7.59 |
| **Total ADL** | **47.90 (17.58)** | **43.0-52.8** | **61.91(24.01)** | **58.63-65.19** |

Mean scores for all six domains of the ADLQ as well as the mean total ADL score were higher among the controls (mean total score=61.9) when compared to the cases (mean total score=47.9).

Table 7.1 summarizes the severity of functional impairment among cases and controls.

***Table 7.1: Frequency Distribution of the Severity of Functional Impairment of Cases and Controls***

| **Functional impairment** | **QOL status** | | | |
| --- | --- | --- | --- | --- |
|  | **Cases (n=64)** | | **Controls (n=208)** | |
|  | **No.** | **%** | **No.** | **%** |
| None-mild impairment | 18 | 28.8 | 34 | 16.3 |
| Moderate impairment | 42 | 65.4 | 86 | 41.3 |
| Severe impairment | 4 | 5.8 | 88 | 42.3 |

Out of all controls, there were 88 (42.3%) with severe functional impairment according to ADLQ scores, whereas only 4 (5.8%) among the cases were categorized as such.

**Neuro-psychiatric symptoms among patients with dementia**

The presence of neuropsychiatric symptoms among dementia patients was assessed using the Neuro-psychiatric Inventory (NPI) ([Cummings, 1997](#_ENREF_2)). It is a validated informant based interview that assesses neuropsychiatric symptoms over the previous month. NPI has 12 items representing 12 neuro-psychiatric domains. The initial domain question has a ‘yes’ (present) or ‘no’ (absent) response. If the answer is ‘yes’, the caregiver is asked to rate the frequency of occurrence on a 4-point scale and the severity of the symptom on a 3-point scale. The domain score is calculated by multiplying the frequency score by the severity score of that particular item. According to Cumming (1994), NPI items are categorized into four subsyndromes as follows; (Table 8).

The total NPI score was obtained by summing up all the individual domain scores. The NPI total score ranged from 1 to 144.

***Table 7: Categorization of NPI items***

| **Sub-syndrome** | **Domains** | **Item No.** |
| --- | --- | --- |
| **Hyperactivity** | Agitation or aggression | 3 |
|  | Irritability | 7 |
|  | Euphoria | 8 |
|  | disinhibition | 9 |
|  | Aberrant motor behaviour | 1 |
| **Affective** | Dysphoria/depressed mood | 4 |
|  | Anxiety | 5 |
|  | Night time behaviour | 11 |
|  | Appetite/eating abnormalities | 12 |
| **Apathy** | Apathy | 6 |
|  | Appetite/eating abnormalities | 12 |
|  | Aberrant motor behaviour | 11 |
|  | Night time behaviour | 10 |
| **Psychosis** | Delusions | 1 |
|  | Hallucinations | 2 |
|  | Anxiety | 5 |

NPI sub-syndrome scores were calculated separately and compared between cases and controls. Table 8.1 summarizes the descriptive statistics of NPI sub-syndrome scores among cases and controls.

Of the four neuropsychiatry sub-syndromes, affective sub-syndrome showed the highest mean difference among cases (mean=3.78) and controls (mean=7.2). Mean score for hyperactivity sub-syndrome among cases (mean=8.0) was slightly higher than that among the controls (mean=7.36).

***Table 8.1 : Descriptive Statistics of the NPI Sub-syndrome Scores among Cases and Controls***

| **NPI**  **Sub- syndrome** | **QOL status** | | | | | |
| --- | --- | --- | --- | --- | --- | --- |
|  | **Cases (n=64)** | | | **Controls (n=208)** | | |
|  | **Mean (SD)** | **95% CI** | **Range** | **Mean (SD)** | **95% CI** | **Range** |
| **Hyperactivity** | 8.0  (3.25) | 7.09-8.90 | 0-13  (13) | 7.36  4.91 | 6.69-8.03 | 0-26  (26) |
|  |  |  |  |  |  |  |
| **Affective** | 3.78  (2.75) | 3.02-4.55 | 1-16  (15) | 7.20  (4.52) | 6.58-7.82 | 0-20  (20) |
|  |  |  |  |  |  |  |
| **Apathy** | 7.63  (3.91) | 6.54-8.72 | 2-13  (11) | 8.43  (5.19) | 7.72-9.14 | 0-28  (28) |
|  |  |  |  |  |  |  |
| **Psychosis** | 1.82  (2.63) | 1.09-2.56 | 0-12  (12) | 2.75  (3.58) | 2.26-3.24 | 0-13  (13) |

The presence of neuropsychiatric symptoms was categorized as follows:

| **Total score** | <20 | Mild |
| --- | --- | --- |
|  | 20-50 | Moderate |
|  | >50 | Severe |

Table 8.2 shows the severity of neuropsychiatric symptoms according to total NPI scores among cases and controls.

Majority of the cases (80.8%) and controls (61.5%) in the study population were having mild neuropsychiatric symptoms.

***Table 8.2: Severity of Neuropsychiatric Symptoms According to Total NPI scores among Cases and Controls***

| **NPI severity** | **QOL status** | | | |
| --- | --- | --- | --- | --- |
|  | **Cases (n=64)** | | **Controls (n=208)** | |
|  | **No.** | **%** | **No.** | **%** |
| Mild | 52 | 80.8 | 128 | 61.5 |
| Moderate | 12 | 19.2 | 75 | 36.1 |
| Severe | 0 | 0 | 5 | 2.4 |

**Caregiver burden**

Caregiver burden was assessed by the validated Sinhala version of Zarit Burden Interview ([Abeywickrema, Weerasundera, & Ranasinghe, 2015](#_ENREF_1)). Primary caregivers were asked to specify the extent of burden experienced while providing care to the patients with dementia. Zarit Burden Interview (ZBI) has 22 items, with responses in a Likert scale, ranging from ‘not at all’ to ‘extremely’. Total score was obtained by summing all the items endorsed. The scores ranged from 0 to 88. Higher the score, more severe the caregiver burden was.

The burden of providing care was categorized as follows;

| **Total score** | 0 -21 | Little or no burden |
| --- | --- | --- |
|  | 21-40 | Mild to moderate burden |
|  | 41-60 | Moderate to severe burden |
|  | 61-88 | Severe burden |

Table 9 shows the severity of caregiver burden among the primary caregivers of cases and controls.

Majority of the caregivers of cases (53.8%) and controls (50%) were having mild to moderate burden according to the ZBI assessment. Only nine caregivers had severe caregiver burden, and all of them belonged to controls (n=9; 4.3%).

***Table 9: Frequency Distribution of the Severity of Caregiver Burden of Primary Caregivers of Cases and Controls***

| **Caregiver burden** | **QOL status** | | | |
| --- | --- | --- | --- | --- |
|  | **Cases (n=64)** | | **Controls (n=208)** | |
|  | **No.** | **%** | **No.** | **%** |
| little or no burden | 24 | 36.5 | 52 | 25 |
| Mild to moderate burden | 34 | 53.8 | 104 | 50 |
| Moderate to severe burden | 6 | 9.6 | 43 | 20.7 |
| Severe burden | 0 | 0 | 9 | 4.3 |

Majority of the caregivers of cases (53.8%) and controls (50%) were having mild to moderate burden according to the ZBI assessment. Only nine caregivers had severe caregiver burden, and all of them belonged to controls (n=9; 4.3%).
